# CYP24A1 Expression Inversely Correlates with Melanoma Progression: Clinic-Pathological Studies

**DOI:** 10.3390/ijms151019000

**Published:** 2014-10-20

**Authors:** Anna A. Brożyna, Cezary Jochymski, Zorica Janjetovic, Wojciech Jóźwicki, Robert C. Tuckey, Andrzej T. Slominski

**Affiliations:** 1 Department of Tumor Pathology and Pathomorphology, the Ludwik Rydygier Collegium Medicum in Bydgoszcz, Nicolaus Copernicus University in Torun, 85-796 Bygoszcz, Poland; E-Mail: jozwickiw@co.bydgoszcz.pl; 2 Department of Tumor Pathology and Pathomorphology, Oncology Centre-Prof. Franciszek Łukaszczyk Memorial Hospital, 85-796 Bygoszcz, Poland; E-Mail: cezaryjochymski@tlen.pl; 3 Departments of Pathology and Laboratory Medicine, University of Tennessee Health Science Center, Memphis, TN 38163, USA; E-Mail: zjanjeto@uthsc.edu; 4 School of Chemistry and Biochemistry, the University of Western Australia, Crawley, WA 6009, Australia; E-Mail: robert.tuckey@uwa.edu.au; 5 Division of Rheumatology, Department of Medicine, University of Tennessee Health Science Center, Memphis, TN 38163, USA

**Keywords:** skin melanoma, vitamin D, CYP24A1

## Abstract

The major role of 24-hydroxylase (CYP24A1) is to maintain 1,25-dihydroxyvitamin D3 (1,25(OH)_2_D3) homeostasis. Recently, it has been discovered that CYP24A1 also catalyses the hydroxylation of 20(OH)D3, producing dihydroxy-derivatives that show very effective antitumorigenic activities. Previously we showed a negative correlation of vitamin D receptor (VDR) and CYP27B1 expression with progression, aggressiveness and overall or disease-free survivals of skin melanomas. Therefore, we analyzed CYP24A1 expression in relation to clinicopathomorphological features of nevi, skin melanomas and metastases. In melanocytic tumors, the level of CYP24A1 was higher than in the normal epidermis. The statistically highest mean CYP24A1 level was found in nevi and early stage melanomas. With melanoma progression, CYP24A1 levels decreased and in advanced stages were comparable to the normal epidermis and metastases. Furthermore, the CYP24A1 expression positively correlated with VDR and CYP27B1, and negatively correlated with mitotic activity. Lower CYP24A1 levels correlated with the presence of ulceration, necrosis, nodular type and amelanotic phenotypes. Moreover, a lack of detectable CYP24A1 expression was related to shorter overall and disease-free survival. In conclusion, the local vitamin D endocrine system affects melanoma behavior and an elevated level of CYP24A1 appears to have an important impact on the formation of melanocytic nevi and melanomagenesis, or progression, at early stages of tumor development.

## 1. Introduction

Calcitriol, also known as 1α,25-Dihydroxyvitamin D3 (1α,25(OH)2D3), regulates a broad range of bodily functions aside from its well-known role in the regulation of calcium levels. 1α,25(OH)2D3 acts through interaction with the nuclear vitamin D receptor (VDR), expressed widely in both normal and malignant tissues. After activation via binding of 1α,25(OH)2D3, VDR forms a heterodimer with retinoid acid X receptor (RXR) which then translocates into the nucleus where it binds to the regulatory regions of target genes, acting as a transcription factor [1,2,3,4]. Under normal conditions the levels of 1α,25(OH)2D3 are precisely regulated. This biologically active form of vitamin D3 is generated by two hydroxylation reactions. The first step is the hydroxylation of vitamin D3 at the carbon 25 (C25) by CYP2R1 or CYP27A1 to form 25-hydroxyvitamin D3 (25(OH)D3, which predominantly occurs in the liver [5]. The second step is the 1α-hydroxylation of 25(OH)D3 to form the 1,25(OH)2D3 which occurs predominantly in the kidney, catalysed by CYP27B1 [3,5,6,7]. 1,25(OH)2D3 is inactivated by CYP24A1 which can oxidize the side chain of 1,25(OH)2D3 (as well as its precursor, 25(OH)D3), by both the carbon 24 (C24)-oxidation pathway producing calcitroic acid, and the carbon 23 (C23)-oxidation pathway producing 1,25-dihydroxyvitamin D3-26,23-lactone [8,9,10,11]. High serum 1,25(OH)2D3 concentrations feedback to stimulate the expression of CYP24A1 as an important part of the mechanism controlling levels of 1,25(OH)2D3 [9,10,11,12,13,14].

High serum levels of 25(OH)D3 correlate with a reduced risk of some cancers, such as prostate, ovarian and breast cancers [15,16,17,18]. A deficiency in 25(OH)D3 is also found in melanoma patients and it correlates with more advanced stages and a worse prognosis [19].

Cytochrome P450 enzymes regulate both systemic and local levels of vitamin D3 and disturbances to this system can deregulate the action of the active form, 1,25(OH)2D3. Besides the kidney, the expression of the activating enzyme, CYP27B1, was found in a range of other normal tissues within the body [20,21]. Its expression is also observed in some tumors, including melanomas [22,23,24,25]. However, under pathological conditions its expression is reduced [22,26,27,28]. It is accepted that CYP24A1, by inactivating 1,25(OH)2D3, attenuates the antitumorigenic activity of the VDR signaling system [2,3,29,30]. Increased levels of CYP24A1 have been observed in some human cancers including colon, breast and lung tumors [22,31,32]. It has been proposed that overexpression of CYP24A1 results in an increased resistance to the antiproliferative action of calcitriol [29,32,33]. In addition, CYP24A1 has been considered as a putative proto-oncogene and its inhibition is proposed as an adjuvant in vitamin-D based cancer treatment [34,35,36].

Previously, we found that decreased expression of VDR and CYP27B1 are associated with progression of melanoma and decreased overall and disease-free survival of melanoma patients [26,37,38], and that novel CYP11A1-derived forms of vitamin D have anti-melanoma activity [39,40]. These findings illustrate that local vitamin D activation and the ability of melanoma cells to respond to active forms of vitamin D are important in melanomagenesis, its progression and therapy [41]. In a continuation of these studies, we have analysed changes in the expression of CYP24A1 during progression of melanocytic tumors and melanomas. To perform these analyses we used 104 cases, including 67 melanomas.

## 2. Results

### 2.1. CYP24A1 Immunostaining in Melanocytic Lesions

**Figure 1:**
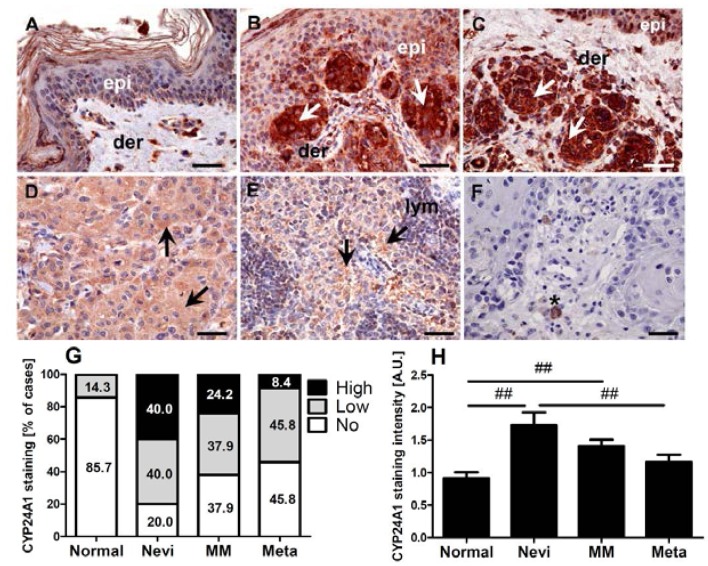
Immunostaining of CYP24A1 in skin. Representative immunostaining is shown for normal skin (**A**); epidermal nevi (**B**); dermal nevi (**C**); melanoma (**D**); lymph node metastasis (**E**) and negative control (**F**); Distribution (**G**) and mean immunostaining (**H**) of CYP24A1 are shown for normal skin, nevi, primary melanoma (MM) and melanoma metastases (meta). Statistically significant differences are denoted with as ## *p* < 0.01 with *t*-test. Arrows show CYP24A1 immunostaining, epi: epidermis, der: dermis, lym: lymphocytes, asterisks: melanin, scale bars: 50 µm.

CYP24A1 immunostaining showed a cytoplasmic localization (Figure 1A–F). In normal skin CYP24A1 was seen in epidermal and dermal cells (melanocytes, keratinocytes, mononuclear dermal cells and fibroblasts). The expression of CYP24A1 was observed in 14.3% of normal skin samples, 80.0% of nevi, 61.4% of melanomas and 54.2% of metastases. High CYP24A1 expression was observed mostly in nevi (40.0% of cases) with its incidence in malignant lesions being decreased (24.2% of primary melanomas and 8.3% of metastases). The incidence of cases with low CYP24A1 in nevi, melanomas and metastases was similar, 40.0%, 37.9% and 45.8% of cases, respectively (Figure 1G).

The mean CYP24A1 immunostaining was the highest in melanocytic nevi. In melanomas the CYP24A1 level was slightly elevated in comparison to the surrounding normal epidermis. In lymph node metastases, the mean CYP24A1 level was comparable to that of normal epidermis (Figure 1H).

**Figure 2:**
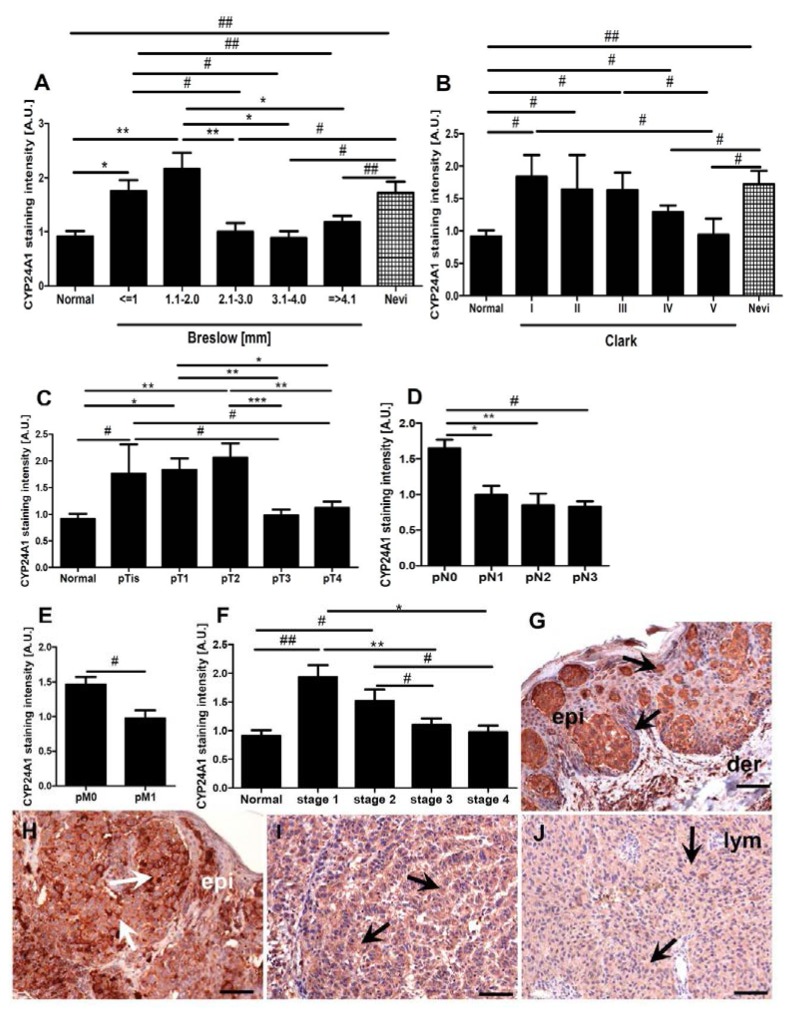
Mean immunostaining of CYP24A1 in normal skin and primary melanomas. Melanomas were stratified according to Clark’s level (**A**); Breslow’s thickeness (**B**); pT stage (**C**); pN (**D**); pM (**E**) and overall stage (**F**); Statistically significant differences are denoted as # *p* < 0.05 and ## *p* < 0.01 with *t*-test and with asterisks as * *p* < 0.05, ** *p* < 0.01 and *** *p* < 0.001 with ANOVA. Representative immunostaining is shown for pTis (**G**); pT2 (**H**); pT3(**I**) and pT4 (**J**) melanomas. Arrows show CYP24A1 immunostaining, epi: epidermis, der: dermis, lym: lymphocytes, scale bars: 100 μm.

Detailed analysis of CYP24A1 immunostaining in melanomas, stratified according to their Breslow’s thickness and Clark’s level, revealed comparable CYP24A1 levels in thin melanomas (Breslow < 2.0 mm and Clark I–II) and melanocytic nevi (Figure 2A,B). However, progression to more advanced stages was associated with a decrease of CYP24A1 expression. Advanced melanomas with a Breslow’s depth > 2.0 mm showed a more pronounced decrease of CYP24A1 than melanomas at III–V Clark levels. However, for melanoma progression evaluated both with Breslow’s thickness and Clark’s level, negative correlation was found for CYP24A1 immunostaining (*r* = −0.3; *p* = 0.011 and *r* = −0.3; *p* = 0.02, respectively). There were no differences in CYP24A1 immunoreactivity between normal epidermis and advanced melanomas and metastases.

A similar relationship was found when melanomas were classified according to the pTNM staging system (Figure 2C,E). In pTis and pT1–2 melanomas, high CYP24A1 immunostaining was more often observed than in pT3–4 melanomas (47.8% and 2.9%, respectively). On the contrary, a lack of CYP24 in pTis and pT1–2 melanomas was observed only in 17.4% of cases and in pT3–4 in 62.3% of cases. The mean CYP24A1 immunostaining was also significantly higher in less advanced (pTis, pT1, pT2) than in more advanced (pT3, pT4) melanomas (Figure 2C). CYP24A1 was also analyzed in relation to lymph node and distant metastases and it was found that melanomas that developed metastases (pN1–3, pM1) had a distinct and significant decrease of CYP24A1 expression in comparison to non-metastasizing ones (Figure 2D,E). Correspondingly, the overall stage of melanomas was inversely correlated with the CYP24A1 immunostaining level (*r* = −0.5, *p* < 0.0001). Melanomas at stages 1 and 2 showed significantly higher CYP24A1 expression than melanomas at stages 3 and 4 (Figure 2F). In the latter, CYP24A1 expression was similar to that observed in normal skin. Representative CYP24A1 immunostaining in melanomas at different pT stage is shown in Figure 2G,J.

### 2.2. Expression of CYP24A1 in Cultured Melanoma Cells, Epidermal Melanocytes and Keratinocytes

*CYP24A1* gene expression was evaluated in 13 human melanoma lines in comparison to second passages of human epidermal melanocytes from neonatuses (HEMn) and human epidermal melanocytes from adult (HEMa), and kuman epidermal keratinocytes from neonatuses (HEKn), human epidermal keratinocytes from adults (HEKa) and immortalized HaCaT epidermal keratinocytes (Table 1). The highest expression of CYP24A1 was seen in neonatal melanocytes and was statistically higher than in 12 melanoma lines, the exception being the YUAME (human melanoma from Yale Institute) line, and higher than in primary and immortalized keratinocytes (Table 1). Expression of the *CYP24A1* gene in human adult melanocytes was lower than in immortalized (HaCaT) keratinocytes, but similar to normal neonatal and adult keratinocytes. However, its expression was lower than in the YUAME melanoma line, at a similar level to expression in the YUCOT and YUKIM (human melanomas from Yale Institute) cell lines, and higher than in the rest of melanomas (10). Expression of the *CYP241* gene in normal neonatal and adult keratinocytes did not differ significantly from melanoma lines. However, expression of the *CYP24A1* gene in immortalized HaCaT keratinocytes was lower than in the YUAME line, at a similar level to expression in the YUKIM line and higher than in the other 11 melanoma lines examined.

**Table 1:** Expression of *CYP24 mRNA* in cultured human (H) melanoma and human epidermal skin cells. A lower crossing point (Cp) value reflects a more abundant signal for each particular gene (PCR product) of interest.

Identification	Cell Type	Mean Cp Value ± SD (Lower Number Means Higher Expression Level)	Statistical Significance (*p* Value) *vs.*					
			HEMn	HEMa	HEKn	HEKa	HaCaT
YUWERA	Human melanoma	10.3 ± 0.2	<0.001	<0.01	NS	NS	<0.001
YUTICA	Human melanoma	12.0 ± 0.4	<0.001	<0.001	NS	NS	<0.001
YUKSI	Human melanoma	17.3 ± 0.3	<0.001	<0.001	NS	NS	<0.001
YULAC	Human melanoma	14.5 ± 0.3	<0.001	<0.001	NS	NS	<0.001
YUSIV	Human melanoma	15.2 ± 0.3	<0.001	<0.001	NS	NS	<0.001
YUAME	Human melanoma	4.6 ± 0.3	NS	<0.01	NS	NS	<0.001
YUROB	Human melanoma	15.2 ± 0.1	<0.001	<0.001	NS	NS	<0.001
YUCOT	Human melanoma	8.3 ± 0.3	<0.001	NS	NS	NS	<0.01
YUKIM	Human melanoma	6.7 ± 0.4	<0.01	NS	NS	NS	NS
YUMUT	Human melanoma	11.6 ± 0.2	<0.001	<0.001	NS	NS	<0.001
YUKOLI	Human melanoma	9.7 ± 0.1	<0.001	<0.01	NS	NS	<0.001
SBCE2	Human melanoma	9.3 ± 0.5	<0.001	<0.05	NS	NS	<0.01
WM1341	Human melanoma, amelanotic	11.3 ± 0.2	<0.001	<0.001	NS	NS	<0.001
HEMn	Epidermal melanocytes, neonatal	4.1 ± 0.1	NA	<0.001	<0.001	<0.01	<0.001
HEMa	Epidermal melanocytes, adult	7.7 ± 0.4	<0.001	NA	NS	NS	<0.01
HEKn	Epidermal keratinocytes, neonatal	9.3 ± 3.0	<0.001	NS	NA	NS	NS
HEKa	Epidermal keratinocytes, adult	8.0 ± 2.7	<0.01	NS	NS	NA	<0.01
HaCaT	Immortalised keratinocytes	6.7 ± 0.07	<0.001	<0.01	NS	<0.01	NA

Cells were grown as described in the Materials and Methods section. Relative gene expression data were calculated using the crossing point (Cp) method. Changes in gene expression are presented as relative quantities using the mean Cp (Normalized target) ± SD to calculate the difference between the target gene and the reference gene by comparing the first cycle of appearance above the threshold (crossing point, Cp) using standardized algorithms in the Roche LC480 1.5 software package. Data are compared using the Student’s *t*-test with significant difference when *p* < 0.05; NS (not significant): *p* > 0.05; NA: not applicable. For human melanomas and HaCaT keratinocytes data were obtained from triplicate assays (*n* = 3). Data for normal keratinocytes and melanocytes are combined from two separate donors with independent cultures run in triplicate (*n* = 6).

In a separate experiment we tested the effect of induction of melanogenesis in SKMel-188 human melanoma cells on *CYP24A1* gene expression. Culturing of cells in media containing tyrosine led to stimulation of melanin pigmentation and a significant increase (*p* < 0.001) in the expression of the *CYP24A1* gene: amelanotic cells (16.24 ± 0.24) *vs.* melanotic cells (14.63 ± 0.16), as evaluated by the Cp method (see Table 1).

### 2.3. CYP24A1 Immunostaining in Relation to Melanoma Pathomorphological Features

**Figure 3:**
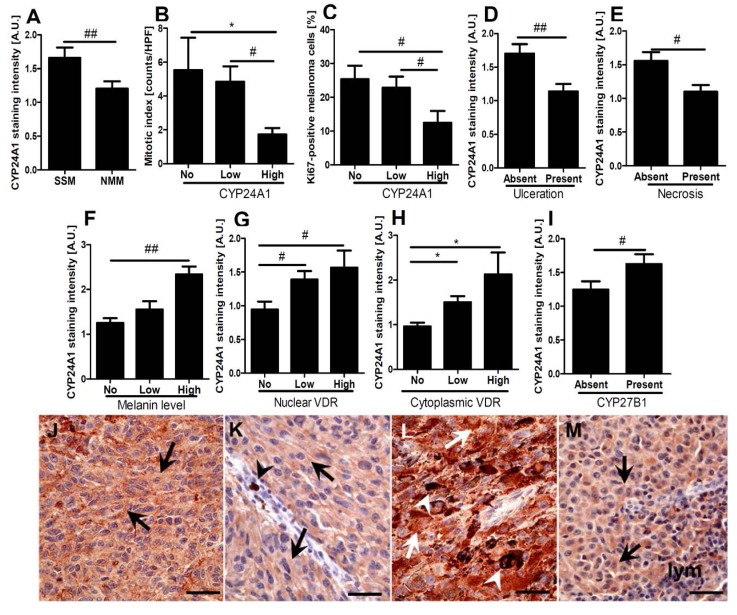
Immunostaining of CYP24A1 in relation to melanoma prognostic markers. Relationship between CYP24A1 immunostaining and histological type (**A**); mitotic index (**B**); Ki-67 (**C**); ulceration (**D**); necrosis (**E**); level of pigmentation (**F**); nuclear (**G**) and cytoplasmic (**H**) vitamin D receptor (VDR) staining intensity (correlation *r* = 0.3, *p* = 0.016 and *r* = 0.5, *p* < 0.001, respectively) and CYP27B1 (**I**) (correlation *r* = 0.3, *p* < 0.03). Statistically significant differences are denoted as # *p* < 0.05 and ## *p* < 0.01 with *t*-test and with asterisks as * *p* < 0.05 with ANOVA. Representative immunostaining of CYP24A1 is shown for superficial spreading melanomas (SSM) (**J**, pT4b, stage 3c, Breslow depth 9 mm) and nodular malignant melanomas (NMM) (**K**, pT4b, stage 2c, Breslow depth 6 mm), pigmented (**L**) and amelanotic (**M**) melanomas. Arrows show CYP24A1 immunostaining, lym: lymphocytes, arrow heads: melanin, scale bars: 50 μm.

To assess the role of CYP24A1 in melanoma biology, behavior and aggressiveness, we analyzed its expression in relation to histological type, proliferation rate, ulceration, tumor infiltrating lymphocytes (TILs), melaninization and solar elastosis. Higher CYP24A1 immunostaining was observed in superficial spreading melanomas (SSM) than nodular malignant melanomas (NMM) (Figure 3A,J,K).

Proliferative activity, assessed by mitotic index counts and Ki-67 immunocytochemistry, showed a negative correlation between CYP24A1 immunostaining and proliferation markers (*r* = −0.3, *p* < 0.05 and *r* = −0.3, *p* < 0.02, respectively). Melanomas with high CYP24A1 immunostaining showed both the lowest mitotic index and the lowest percentage of Ki-67-positive melanoma cells (Figure 3B,C). Importantly, higher CYP24A1 expression was found in non-ulcerated melanomas (Figure 3D). Similarly, melanomas without necrosis also showed significantly higher CYP24A1 immunostaining in comparison to melanomas with necrosis (Figure 3E).

We also found a positive correlation between melanin pigmentation and CYP24A1 expression (*r* = 0.3, *p* < 0.005), with significantly higher CYP24A1 in highly pigmented melanomas than in amelanotic or moderately pigmented tumors ( Figure 3F,L,M) (*p* < 0.003). This correlation is consistent with cell culture data showing that strongly pigmented cells express higher mRNA levels of CYP24A1 (see 2.2). No relationship was found between CYP24A1 immunostaining and TILs or solar elastosis.

### 2.4. Correlations between CYP24A1, Vitamin D Receptor (VDR) and CYP27B1 Immunostaining

Both nuclear and cytoplasmic VDR expression positively correlated with CYP24A1 immunostaning that was the highest in tumors expressing highest levels of VDR, and the lowest for tumors having VDR below detectability level (*r* = 0.3, *p* < 0.02 and *r* = 0.5, *p* < 0.0005, respectively) (Figure 3G,H). A similar correlation was observed for CYP24A1 and CYP27B1 expression levels (*r* = 0.3, *p* < 0.03) (Figure 3I).

### 2.5. Correlation between CYP24A1 Immunostaining and Overall Survival

**Figure 4:**
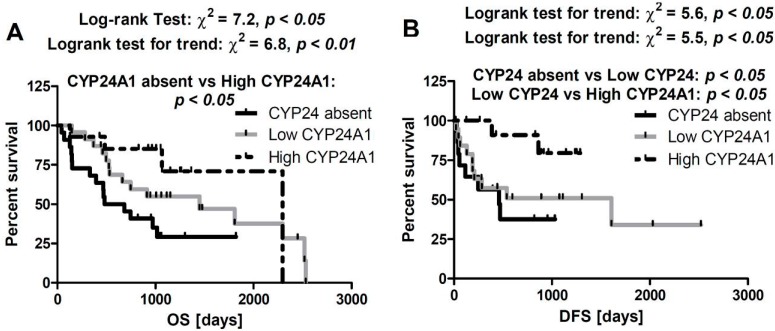
Correlation of overall survival time (**A**) and disease-free survival time (**B**) with the relative expression of CYP24A1.

We found significant shortening of overall survival time (OS) for patients without CYP24A1 in primary melanomas (581 days *vs.* 1449 days for melanomas with low CYP24A1, and 2297 days for melanomas with high CYP24A1) (Figure 4A). These differences were more evident in early-stage melanomas (pTis–pT2). Similarly, disease-free survival time (DFS) was the highest in melanomas with high CYP24A1 in comparison to melanomas with low or absent CYP24A1 (1607, 539, 459 median days, respectively) (Figure 4B). CYP24A1 expression in lymph node metastases neither influenced OS nor DFS.

## 3. Discussion

In this study we show an unexpected pattern of CYP24A1 expression in human skin in respect to normal and cancerous states. Thus, CYP24A1 expression in normal epidermis was lower than in melanocytic nevi and early-stage melanomas (pTis–pT2), while being similar to pT > 2 and metastatic melanomas. In melanomas, the CYP24A1 level was negatively correlated with Brelow’s thickness, Clark’s level and the overall stage. In addition, melanomas with lymph node and distant metastases also had significantly lower CYP24A1 levels than non-metastasizing melanomas. CYP24A1 was also reduced in melanomas with more aggressive phenotypes, as defined by high proliferative activity, ulceration and nodular type. Higher CYP24A1 levels were accompanied by higher CYP27B1 and VDR levels. Furthermore, shorter OS and DFS were found in melanomas without detectable CYP24A1.

Theoretically, elevated levels of CYP24A1 should stimulate degradation of 1,25(OH)_2_D3 with attenuation of its antitumorigenic actions, enabling tumor growth or progression [11,29]. An increased level of CYP24A1 was observed in lung, ovarian, colon, esophageal and renal cancers [42,43,44]. In lung cancers, elevated expression of CYP24A1 was more prominent in poorly differentiated tumors and was correlated with worse survival prognosis. Also, a lung cancer cell line with high CYP24A1 expression was also more resistant to the antiproliferative action of vitamin D3 than cell lines with low CYP24A1 expression [45]. Similarly, esophageal cancer patients had better prognosis when the expression of CYP24A1 was lower, and the esophageal cancer cell lines with higher CYP24A1 expression showed a higher proliferation rate when compared to cell lines without CYP24A1 [44]. In colonic carcinomas, a positive correlation between CYP24A1 expression and Ki-67 was reported [46]. Moreover, inhibition of CYP24A1 has been proposed as an adjuvant approach to increase the antitumor activity of vitamin D [34].

In contrast to the above reports, in our cohort of melanoma patients an increased level of CYP24A1 was observed in benign melanocytic tumors and early-stage melanomas, while in advanced tumors, which developed metastases and lymph node metastases, the level of CYP24A1 was low and comparable to the level observed in normal skin. Also, the lack of CYP24A1 expression in melanomas was accompanied by a higher proliferation rate and unfavorable prognostic histopathological markers (ulceration, necrosis, nodular type melanoma). A similar trend was observed in thyroid pathology where increased CYP24A1 expression was found in follicular adenomas, while in differentiated thyroid cancers its level decreased, being comparable with normal tissues [47]. Thyroid cancers with lymph-nodes and distant metastases had lower CYP24A1 expression in comparison to non-metastasizing cancers, and the lack of CYP24A1 in anaplastic thyroid cancer was accompanied by a higher proliferation rate [47]. In addition, in breast cancers CYP24A1 expression decreased during tumor development [42]. Thus, our findings on the correlation between tumor pathology and/or progression markers are in agreement with reports on breast and thyroid cancers. Furthermore, the clinical outcome correlated well with pathology, e.g., OS in CYP24A1-negative melanomas was significantly shorter in comparison to melanoma expressing high CYP24A1, and DFS was positively correlated with higher expression of CYP24A1.

To further analyze this surprising inverse correlation between CYP24A1 expression and melanoma progression, we compared *CYP24A1* gene expression in cultured normal neonatal or adult epidermal melanocytes with 13 melanoma lines. We found that the majority of melanoma lines had lower expression of the *CYP24A1* gene than neonatal melanocytes (except 1 line) and adult melanocytes (except 3 lines). This is consistent with lower expression of CYP24A1 in advanced melanomas in comparison to melanocytic nevi, discussed above. Similarly to the immunohistochemical analysis showing comparable expression of CYP24A1 in epidermis and advanced melanomas, *CY24A1* gene expression between either neonatal or adult epidermal keratinocytes did not differ significantly from all cultured melanoma lines. Thus, *in vitro* analysis shows a similar trend for CYP24A1 expression to the immunocytochemical analyses of pathological skin.

This surprising expression pattern of CYP24A1 in human melanomas could in part be explained by the recent discovery that CYP24A1, apart from inactivating calcitriol, also hydroxylates 20-hydroxyvitamin D3 (20(OH)D3) to two major dihydroxy-derivatives, 20,24-dihydroxyvitamin D3 (20,24(OH)_2_D3) and 20,25-dihydroxyvitamin D3 (20,25(OH)_2_D3), which are significantly more efficient at inhibiting melanoma growth in soft agar than 1,25(OH)_2_D3 and 20(OH)D3 [48]. Note, that 20(OH)D3 is the first and a major metabolite of the newly discovered pathway of vitamin D activation initiated by CYP11A1, a pathway that operates in the skin [49,50,51,52]. The products of this pathway are biologically active [52], including exhibiting anti-melanoma activity [40,53]. They also act on alternative receptors to the VDR [54]. In addition, it should be noted that products of classical vitamin D metabolism including 24,25(OH)_2_D3 [55] or others [56] show biological activity, apparently acting through alternative receptors [57].

Additional histopathological and immunocytochemical analyses gave a positive correlation between CYP24A1 expressions and the expression of VDR, CYP27B1 and melanin content. The positive correlation between expression of CYP24A1, VDR and CYP27B1 is consistent with upregulation of CYP24A1 by 1,25(OH)2D3 (product of CYP27B1 activity) through activation of the VDR [11]. The higher expression of CYP24A1 in strongly pigmented melanomas was a rather unexpected finding since our previous studies have shown a negative correlation between VDR and CYP27B1 expression [26,37,39]. However, changes in CYP24A1 in relation to melanin content are consistent with a regulatory function of melanogenesis or its intermediates on biological properties of melanoma cells [58,59,60].

## 4. Materials and Methods

### 4.1. Patients

The expression of CYP24A1 was evaluated in 104 tissue samples including 7 normal skin samples, 15 nevi, 57 skin melanomas and 25 metastases, obtained from 67 patients treated in the Oncology Centre in Bydgoszcz. Normal skin samples were obtained from patients who underwent surgical operations not related to skin diseases. The clinico-pathomorphological characteristics of patients are presented in Table 2. The study was approved by the Committee of Ethics of Scientific Research of Collegium Medicum of Nicolaus Copernicus University, Poland (approval no. KB 448/2009 from October 2009).

**Table 2:** Patient and melanoma characteristics.

Clinicopathologic Features		No.
Type of Lesions	All samples	104
	Nevi	15
	Primary melanomas	57
	Nodular	31
	Superficial spreading	26
	Melanoma metastases	25
	Normal skin	7
Age (year)	Nevi: mean = 43, median = 36; range = 20–85	15
	Melanomas: mean = 60, median = 57; range = 25–100	57
Patients’ Gender	Nevi: M/F	5/10
	Melanomas: M/F	28/29
Anatomical Site	Nevi	15
	Extremity	2
	Head and neck	3
	Trunk	10
	Melanoma	57
	Acral	2
	Anogenital	2
	Extremity	19
	Head and neck	12
	Trunk	22
Breslow Thickness (mm)	0 (is)	3
	0–1	13
	1.1–2	6
	2.1–3	9
	3.1–4	4
	>4.0	22
Clark Level	I	7
	II	3
	III	8
	IV	32
	V	7
pT	pT0	3
	pT1	13
	pT2	7
	pT3	14
	pT4	20
pN	pN0	29
	pN1	13
	pN2	5
	pN3	9
pM	pM0	50
	pM1	7
Overall Stage	0	3
	1	13
	2	14
	3	20
	4	7

### 4.2. Immunohistochemistry Staining and Evaluation

CYP24A1 immunocytochemistry was performed on 4 µm sections of tissue samples that were formalin-fixed and paraffin-embedded as described previously [26]. Briefly, after antigen retrieval endogenous peroxidase activity was blocked with 3% H_2_O_2_ (Avantor Performance Materials Poland S.A., Gliwice, Poland), followed by permeabilization in 0.2% Triton-X100 (Sigma-Aldrich Co. LLC, St. Louis, MO, USA). Samples were then stained overnight at 4 °C with primary monoclonal mouse anti-CYP24A1 antibody (Abcam, Cambridge, UK) diluted 1:40 in antibody diluent (Dako, Carpinteria, CA, USA). After washing, sections were incubated with secondary anti-mouse antibody EnVision /HRP (Dako, Carpinteria, CA, USA). CYP24A1 antigen was then visualized with ImmPACT NovaRED (Vector Laboratories Inc., Burlingame, CA, USA). After counterstaining with hematoxylin, sections were dehydrated and mounted in permanent medium (Consul Mount; Thermo Fisher Scientific Inc. Waltham, MA, USA). The kidney was used as a positive control.

Immunohistochemical assessments of stained sections were performed by 2 observers (Anna A. Brożyna and Cezary Jochymski) in a blind manner without knowing the histopathological diagnosis or the malignancy grade and other clinical data. CYP24A1 immunostaining was scored semiquantitatively, as follows: SQ = mean (IR × SI)/100, where IR is the percentage of immunoreactive cells and SI is the staining intensity from 0 to 3 arbitrary units (A.U.), either negative (0), weak (1), moderate (2) or strong (3). Staining intensity was evaluated with reference to immunostaining of kidney cells, scored as strong. Cases were then stratified according to the CYP24A1 SQ-score as follows: SQ 0.0–1.0 = no CYP24A1, SQ 1.1–2.0 = low CYP24A1, SQ 2.1–3.0 = high CYP24A1.

Evaluation of VDR, CYP27B1 and Ki-67 immunostaining, mitotic index and melanin content were performed as previously described [26,37,38,39,61,62].

### 4.3. Cell Culture Studies

Human melanoma cells identified with the first two letters YU (gift of Ruth Halaban from Yale University) were cultured in Opti-Mem (Life Technologies, Grand Island, NY, USA) media supplemented with 10% FBS (Fetal Bovine Serum, Atlanta Biologicals, Norcross, GA, USA) [63]. WM1341 and SBCE2 melanoma cells (gift of Meenhard Herlyn from the Wistar Institute) were cultured in DMEM (Dulbecco’s Modification of Eagle’s Medium, Corning Cellgro, Manassas, VA, USA) media supplemented with 10% FBS [40]. Skin cells were isolated from adult skin or neonatal foreskins, as described previously [40,64]. Keratinocytes were cultured in Keratinocyte Growth media (KBM) media supplemented with Keratinocyte Growth factors (KGF) (Lonza, Walkersville, MD, USA), and melanocytes were cultured in Melanocyte Growth media (MBM) media supplemented with Melanocyte Growth factors (MGF) (Lonza, Walkersville, MD, USA). RNA was harvested from cells using the Absolutely RNA Miniprep kit (Stratagen, Agilent Technologies, La Jolla, CA, USA), as previously described [40]. To induce production of melanin pigment in SKMEL-188 cells, Ham’s F10 medium was supplemented with 400 µM of l-tyrosine, while control amelanotic cells were grown in Ham’s F-10 medium (Corning Cellgro, Manassas, VA, USA) for 3 days [60,65].

### 4.4. Reverse Transcription (RT) and Quantitative Polymerase Chain Reaction (qPCR)

Reverse transcription was performed using the Transcriptor First Strand cDNA Synthesis kit (Roche, Mannheim, Germany) using 0.5 µg of RNA per reaction. Primers were designed using the Universal Probe Library software (UPL Roche, Mannheim, Germany): *Cyp24*: l-5'-CATCATGGCCATCAAAACAAT-3', *R*-5'-GCAGCTCGACTGGAGTGAC-3'; *cyclophilin B*: l-5'-TGTGGTGTTTGGCAAAGTTC-3', *R*-5'-GTTTATCCCGGCTGTCTGTC-3'. Real-time PCR (qPCR) data were generated from input cDNA using a TaqMan Master Mix (*n* = 3) that was amplified using standard settings on a Roche LC480 LightCycler (Roche, Mannheim, Germany). Gene expression (expressed as crossing point values, Cp) was normalized using cyclophilin B by the delta-delta-Ct (DDCp) method. Changes in gene expression are presented as relative quantities based on the mean difference between the target gene and the reference gene in the cycle of appearance in time ± SD [60]. It should be noted that the lower the number, the higher the relative level of gene expression. Since the Cp value represents the cycle at which the signal is first detected above a threshold, increases in the relative abundance of the PCR product detected are reflected by a lower Cp numeric value for the gene (PCR product) of interest. Data were compared between melanoma and skin cells using the Student’s *t*-test.

### 4.5. Statistical Analysis

Statistical analyses were performed using Prism 5.0 (GraphPad Software, San Diego, CA, USA). The differences between analyzed categorical variables were compared using a *t*-test or one-way ANOVA. The correlation was evaluated with Pearson’s correlation. Survival analysis was performed using the Log-rank test. Values with *p* < 0.05 were considered as statistically significant.

## 5. Conclusions

In summary, based on our previous studies and the present results, we conclude that the local vitamin D endocrine system affects melanoma behavior in a complex manner and elevated levels of CYP24A1 appear to have an important impact on the formation of melanocytic nevi and melanomagenesis or melanoma progression.
